# Towards a Greater Professional Standing: Evolution of Pharmacy Practice and Education, 1920–2020

**DOI:** 10.3390/pharmacy7030098

**Published:** 2019-07-20

**Authors:** Benjamin Y. Urick, Emily V. Meggs

**Affiliations:** Eshelman School of Pharmacy, University of North Carolina, Chapel Hill, NC 27599, USA

**Keywords:** history of pharmacy, 20th century history, 21st century history, community pharmacy services, pharmacy education

## Abstract

The history of community pharmacy in America since the 1920s is one of slow progress towards greater professional standing through changes in pharmacy education and practice. The history of American community pharmacy in the modern era can be divided into four periods: 1920–1949 (Soda Fountain Era), 1950–1979 (Lick, Stick, Pour and More Era), 1980–2009 (Pharmaceutical Care Era), and 2010–present (Post-Pharmaceutical Care Era). As traditional compounding has waned, leaders within community pharmacy have sought to shift focus from product to patient. Increasing degree requirements and postgraduate training have enhanced pharmacists’ ability to provide patient care services not directly associated with medication dispensing. However, the realities of practice have often fallen short of ideal visions of patient-focused community pharmacy practice. Positive trends in the recognition of the impact of community pharmacists on healthcare value and the need for more optimal medication management suggest that opportunities for community pharmacists to provide patient care may expand through the 21st century.

## 1. Introduction

As long there has been a belief in the medicinal properties of natural substances, there have been people whose duty it was to transform these materia medica into medicines. By the 1800s, however, this traditional role of pharmacy had begun to change. The Industrial Revolution led to mass manufacture of medicinal products which once only the pharmacist could produce. Additionally, new medicines were being discovered which could not be easily derived from traditional materia medica. As traditional compounding began to wane and proprietary products began to replace those which the pharmacist used to make himself, merchandising in pharmacies began to increase. The erosion of traditional roles led to a crisis of professionalism within American community pharmacy, requiring the profession to rethink its role in society. It is with this backdrop that the modern era of community pharmacy in the United States begins.

For this narrative review, the history of American community pharmacy in the modern era can be divided into four periods: 1920–1949 (Soda Fountain Era), 1950–1979 (Lick, Stick, Pour and More Era), 1980–2009 (Pharmaceutical Care Era), and 2010–present (Post-Pharmaceutical Care Era). A slow march towards greater patient care and higher professional standing can be observed across each of these periods as the profession of pharmacy has struggled with what defines community pharmacy and how community pharmacy adds value to society.

## 2. 1920–1949: Soda Fountain Era

### 2.1. Education

By the time the modern era of pharmacy dawned in the 1920s, pharmacy education was rapidly adopting three and four-year degrees as the standard for education [1]. Old-fashioned short courses, designed as supplements to apprenticeship, were falling out of favor and would soon be made obsolete. Pharmacy education in the early part of the 20th century was guided by *The* Pharmaceutical Syllabus [1]. This detailed guide to pharmacy education, created by the American Association of Colleges of Pharmacy (AACP), helped to standardize degree programs as training became more formalized.

The first major study of pharmacy practice, Basic Material for a Pharmaceutical Curriculum, was published in 1927 [2]. This study sought to revise the curriculum contained in the Pharmaceutical Syllabus by developing a new curriculum based on the functional needs of the pharmacy profession [2]. Reflecting the makeup of the profession in the 1920s, the Pharmaceutical Curriculum was focused solely on the needs of pharmacists working in retail settings. The report focused on many areas of study deemed essential to pharmacy practice at the time, including basic sciences of chemistry and physics; medicine-related subjects such as pharmacognosy, botany, pharmacology, physiology, and public health; and practice-related subjects such as small-scale pharmaceutical manufacturing, prescription filling, and retail sales operations. The Pharmaceutical Curriculum did not, however, include any information on diagnosis and treatment of disease. While the authors acknowledged that a pharmacist has a duty to assist their “customers” who have questions on “the cure for an ailment,” they were concerned that too much education would lead to counter-prescribing—dispensing pharmaceuticals to treat a disease without or contrary to a prescription from a physician.

Merchandising and commercial aspects of pharmacy practice were only begrudgingly included in the Pharmaceutical Curriculum. It was acknowledged that merchandising and commercial interests were rampant within the community pharmacy practice. However, one goal of creating a standardized curriculum was to raise professional standards and train more professionally-oriented graduates who were better able to engage with other healthcare practitioners [1]. As such, it was thought that inclusion of merchandising and commercial interests would undermine pharmacy’s professionalism and these aspects of pharmacy practice were excluded from the Pharmaceutical Curriculum. Aided in educational transformation during the Soda Fountain Era was the founding of the Accreditation Council for Pharmaceutical Education (ACPE) in 1932. The ACPE established the first national standards for pharmacy degree program accreditation and, as a result, by 1941, 64 out of 67 colleges of pharmacy had adopted a four-year degree standard.

The educational change began in The Pharmaceutical Syllabus was furthered by the Pharmaceutical Survey which was commissioned by the American Council on Education in 1946 [3]. The Pharmaceutical Survey recognized the growing tension between pharmacists as distributors of mass manufactured products and pharmacists as healthcare professionals. The distribution and merchandising roles were seen as undermining pharmacy’s professionalism. Additionally, the four-year degree was thought to be too short a course of study for the pharmacist to complete a general education as well as a pharmacy education [4], and did not “confer the status that is desired by pharmacists, particularly those who work in rather intimate professional association with physicians, dentists, and members of other health professions who hold professional doctor’s degrees. [4]”

Therefore, to provide a complete education and firm the professional foundation of pharmacy practice, the report recommended the establishment of a six-year Doctor of Pharmacy program to afford “new opportunities for raising the level of preparation for the professional areas of pharmacy [5].” However, the recommendation to lengthen the curriculum was met with opposition by pharmacy educators, and the majority of pharmacy school deans at the time favored the status quo [4]. The debate within the American Association of Colleges of Pharmacy about degree standards would result in substantial changes to pharmacy education in the 1950s.

### 2.2. Practice

As compounding waned, pharmacy in the 1920s found itself questioning its own professional standing. This is reflected in practice as well as education. Concurrently, the enactment of national prohibition in 1919 was a boon to pharmacies’ front-end commercial interest in two major ways [6]. First, the sale and consumption of “medicinal” alcohol was allowed and this created a legal loophole which many pharmacists and physicians exploited. Second, soda fountains became very popular destinations for those seeking alcohol-alternatives. Neither was considered a “professional” activity, but both were surely profitable.

Accordingly, traditional prescription compounding and dispensing became a minor part of pharmacy operations in the 1920s and 1930s. Although 75% of prescriptions still required some compounding [7], less than 1% of pharmacies of pharmacies had more than 50% of their sales from prescription drugs [8]. Even when drugs were dispensed, ethical standards at the time limited pharmacists’ engagement with patients. For example, the 1922 American Pharmaceutical Association (APhA) Code of Ethics [9] stated that:

“[The pharmacist] should never discuss the therapeutic effect of a Physician’s prescription with a patron nor disclose details of composition which the Physician has withheld, suggesting to the patient that such details can be properly discussed with the prescriber only”.

In the 1920s, the transition away from compounding and towards premanufactured proprietary products led to a crisis within the community pharmacy—pharmacy’s traditional role was waning, and it was not clear what the role of a pharmacist was, if not, compounding. The answer, in many ways, was to increase front-end commercial interests through expanding soda fountains and other goods for purchase. Prescription dispensing was essential to the identity of the pharmacy, but was de-emphasized as a part of the pharmacy’s business. This would change as advances in pharmaceutical research in the mid-20th century led to an explosion of new prescription drug products.

## 3. 1950–1979: Lick, Stick, Pour and More Era

As the patient care roles of the pharmacist and educational standards increased from the 1950s through the 1970s, the highest professional activity was no longer dispensing, as it was in the 1920s. The provision of patient care services replaced dispensing as the highest professional activity. This created a cultural shift within community pharmacy practice—and gave rise to the tension between dispensing and patient care which persists into the 21st century. Arguments over the degree needed to support this new version of professionalism were heated, and would not be ended until the 1980s.

### 3.1. Education

The recommendations of the Pharmaceutical Survey laid the foundation for changes to pharmacy education throughout the 1950s, 60s, and 70s. Leaders in pharmacy education acknowledged that the four-year degree was insufficient for the level of training needed to become a pharmacist. There was strong resistance, however, to a mandatory professional doctorate as the entry level practice degree. An uneasy compromise was made with the adoption of a five-year degree standard, despite specific recommendations against the degree from the Pharmaceutical Survey.

As clinical pharmacy and the desire for higher professional standing began to permeate throughout the profession in the 1960s and early 1970s, the movement towards a degree which provided the appropriate professional foundation for clinical pharmacy accelerated. The first pharmacy program to adopt an all-Doctor of Pharmacy (PharmD) standard was the University of Southern California in 1950 [10]. Other programs followed USC’s lead, and by the mid-1970s there were 20 PharmD programs.

Through its emphasis on clinical education and experience, proponents of the PharmD redefined professionalism as not just an avoidance of merchandising or commercial endeavors, but also de-emphasized medication dispensing as a professional activity befitting a PharmD-trained pharmacist. Indeed, dispensing was called a “temporary obfuscation” of the clinical objective of the profession [10]. Educational changes associated with the clinical pharmacy movement also re-emphasized the practice component of pharmacy education, reducing educational focus on theory-based training in basic sciences [11].

### 3.2. Practice

By 1950, the percent of prescriptions which were compounded had fallen to 25% [7]. This percentage would decrease further to less than 5% by 1960 and 1% by 1970 [7]. Concurrent with decreases in traditional compounded prescriptions was a large increase in the number and diversity of premanufactured drug products. An explosion of newly discovered drugs led to an increase of over 50% in the number of drugs dispensed during the 1950s [6]. By the mid-1950s, pharmacists were stepping away from soda fountain and were back behind the pharmacy counter. However, the role had changed substantially from the 1920s. Pharmacists were primarily dispensing premanufactured capsules and tablets, and ethical standards at the time still prohibited them from discussing the contents of prescriptions with patients [12]. Prescription labels from that era commonly omitted the name of the product dispensed—with the idea that labeling the vial with the name of the drug would violate the physician-patient relationship. This was the origin of the modern “lick, stick, and pour” pharmacy practice.

This new version of pharmacy irked many patient care-oriented pharmacists at the time because they desired to do more than simply dispense a product. Eugene White, among the more well-known visionaries of what would become patient care-oriented community pharmacy practice, began working in 1950 at a typical retail-oriented pharmacy. He quickly became disillusioned with practice standards at the time, saying, “After five months of selling lawn seed and paint, cutting glass for window frames, and dispensing a few prescription orders in between, I could longer take it… [13]” White purchased his own pharmacy in 1957 and in 1960 completely transformed his pharmacy into what he termed a “pharmaceutical center.” Gone was the soda fountain and self-serve retail space for candy, stationary, billfolds, toys, and gifts. He added a record system to keep track of families’ prescriptions. He hired a receptionist to greet patients when they entered and built a semi-private patient counseling area. His model even served as the basis for professional pharmacies promoted and designed by McKesson in the mid-1960s [14].

Also innovating during this era were pharmacists in community pharmacy practice settings like the Indian Health Service, which in 1966 required private patient counseling areas in all new pharmacies [15]. The 1960s also witnessed the birth of clinical pharmacy services, with major innovation stemming from experiments including the Ninth Floor Project led by University of California, San Francisco School of Pharmacy faculty [16]. This project revolutionized the provision of pharmacy services in hospitals by building a satellite pharmacy to dispense unit-dose medications specific to each order and to prepare admixtures by pharmacists instead of nurses, which at the time were radical advancements. In addition, the pharmacist was available for consultation on drug information and other clinical questions as they arose. This experiment spurred the development of similar clinically-focused pharmacy roles nationwide would substantially influence changes in pharmacy practice philosophy in the 1980s.

Eugene White and innovators like him replaced customers with patients, and through this they redefined how a professional community pharmacy operated. Combined with the growth in clinical pharmacy in hospitals during this same time, this transformation revolutionized how the profession saw itself. This was change was reflected in the 1969 revision to APhA’s Code of Ethics [17] which referred to a pharmacist’s duty to his patient in the first section:

A pharmacist should hold the health and safety of patients to be of first consideration; he should render to each patient the full measure of his ability as an essential health practitioner.

Practice changes continued through the 1970s. Products became more diverse, and spillover from the clinical pharmacy movement began to expand the array of non-dispensing services provided in pharmacies. Additionally, the first computer systems in the 1970s expanded pharmacists’ abilities to keep dispensing records and check for drug–drug interactions [6].

The period from the 1950s to 1970s was a pivotal time for American pharmacy. The emphasis on front-end merchandising and soda fountains waned as dispensing increased. A new version of professionalism had started to arise within community pharmacy—one focused not on dispensing alone but on dispensing as a part of care for a patient’s medication-related needs. Education also witnessed a similar evolution, with new PharmD degree programs supporting the training needed to provide robust patient care services. Ethical standards likewise evolved, with the 1969 APhA Code of Ethics calling for pharmacists to engage in activity that would have been ethically suspect under previous codes. These changes built the foundation for changes in the 1980s that would further propel the notion that community pharmacists had an obligation to their patients which extended beyond simple dispensing of products.

## 4. 1980–2009: Pharmaceutical Care Era

### 4.1. Education

The final major change to pharmacy education in the last 100 years was the transition from the five-year, entry-level B.S. degree with the optional post-graduate PharmD training to the PharmD becoming the entry level degree. Echoing the Pharmaceutical Survey from nearly 40 years prior, the Final Report of the Task Force on Pharmacy Education, commissioned by the American Pharmaceutical Association (APhA) and released in 1984, called for a universal six-year PharmD degree [18]. Following this call, the universal PharmD degree was put to a vote in the American Association of College of Pharmacy (AACP) House of Delegates in 1985 but was defeated by a narrow margin. Nevertheless, major national conferences and academic papers throughout the end of the 1980s helped sway opinion towards a universal PharmD and acceptance of pharmacy as a clinical profession which needed a professional doctorate. In 1989, A Declaration of Intent was made by ACPE to adopt the PharmD as the universal standard for pharmacy education as soon as 2000 [19,20]. Through the early 1990s, APhA, the National Association of Retail Druggists, and the American Society of Hospital Pharmacists, the American College of Clinical Pharmacy, the National Association of Boards of Pharmacy, and AACP came to actively support the single degree standard but the organization representing chain pharmacies, the National Association of Chain Drug Stores, continued to oppose the concept [20,21,22]. On schedule, the last student to enter an accredited BSPharm program enrolled in 2000 and the transition to a universal PharmD was completed in 2005 [23].

The most evident education-related aspect of the patient care movement within community pharmacy was the development of the first community pharmacy residency programs in the mid-1980s. Training on the provision of clinical services was an explicit goal of these first community pharmacy residencies [24], and this focus has expanded over time [25]. Community pharmacy residency sites have often served as laboratories for advanced practice, requiring community pharmacists to engage in practice-based research and expanding the scope of services offered [26]. The development of formal postgraduate training focused on patient care in community pharmacy settings, combined with a universal PharmD, created a strong foundation for the expanded delivery of patient care services in community pharmacies.

### 4.2. Practice

The 1980s witnessed major change to the philosophy of practice as pharmacy leaders considered pharmacy’s role in the 21st century. Many of these changes were, however, aspirational with some innovative pharmacies leading the way and most lagging behind. In many ways, the movement to bring clinical pharmacy into the community setting was seen as idealistic and out-of-touch with the busy community pharmacy work environment [27].

Nevertheless, the declaration of pharmacy as a clinical profession at the 1985 Hilton Head Conference [28] and the conceptualization of pharmacists’ duties vis-à-vis pharmaceutical care in 1989 through a presentation at the Second Hilton Head Conference [29] and a seminal paper entitled “Opportunities and Responsibilities in Pharmaceutical Care” [30] were a boost to those seeking a greater patient care orientation for community pharmacy practice. Hepler and Strand’s definition of pharmaceutical care placed patient care at the center of pharmacy practice:

Pharmaceutical care is the responsible provision of drug therapy for the purpose of achieving definite outcomes that improve a patient’s quality of life. These outcomes are (1) cure of a disease, (2) elimination or reduction of a patient’s symptomatology, (3) arresting or slowing of a disease process, or (4) preventing a disease or symptomatology.

These changes to practice philosophy were boosted when the federal government in 1990 required prescription counseling as a part of the Medicaid program [31]. The combination of these elements enthusiastically propelled community pharmacy into the last decade of the 20th century. Several pilot projects in the 1990s demonstrated that pharmacists could provide and be remunerated for pharmaceutical care services, which would later be rebranded as medication therapy management (MTM) in the 2000s. The Minnesota Pharmaceutical Care Demonstration Project [32], private and public initiatives in Iowa [33], and the Asheville Project [34], for example, are well-known examples of projects which paid community pharmacists for non-dispensing related services. Training programs, like that of the Iowa Center for Pharmaceutical Care [33], were used nationally and internationally to prepare pharmacists to provide services tested through these demonstration projects. By the end of the 1990s, the market for pharmaceutical care services was sufficiently well-developed to support the launch of OutcomesMTM^®^ in 1999.

The progress of pharmaceutical care era culminated in 2003 with the passing of the Medicare Prescription Drug, Improvement, and Modernization Act (MMA). This act created the Medicare Part D benefit, the first Medicare benefit for retail prescription drugs. The MMA required Part D plan sponsors to include MTM as a part of their benefit structure. Pharmacy viewed MTM as a major victory—finally, the federal government was recognizing the need for more optimal medication use and creating a payment mechanism for it. The enthusiasm was short-lived, however, as the benefit allowed a variety of non-pharmacist providers to deliver MTM, and many Part D plan sponsors sought cost-minimal ways to offer the mandatory benefit.

Also growing during the 1990s was the new role of pharmacists as immunizers. The first immunization training programs were developed by state and national pharmacy organizations in the mid-1990s [35]. By 2003, 34 states allowed pharmacists to provide immunizations but many restrictions were placed on pharmacists’ immunization-related scope of practice [36], but by the end of the decade, all states allowed pharmacists at least some level of immunization authority, and many of the earlier restrictions had been lifted [37]. Pharmacists were critical vaccine distribution sites during the response to the 2009-10 H1N1 influenza pandemic [38] and research from the time suggests that this expanded role had a positive impact on public health [39]. Pharmacist-provided immunizations expanded the total number of patients who were vaccinated, not just shift sites of care from primary care offices to pharmacies.

Despite this momentum, the National Pharmacy Workforce Survey found that in 2004 only 5–10% of community pharmacists reported that their pharmacy provided MTM services [40]. Other services such as health screenings, immunizations, and smoking cessation were more common, but none of these enhanced services were provided at the majority of pharmacies where respondents worked. The 2009 National Pharmacy Workforce Survey, delivered after the full implementation of Medicare Part D, found slightly higher engagement in patient care services, but pharmacists still only spent 8–11% of their time providing patient care services and 70–78% of their time dispensing [41].

As the 21st century dawned, the long-awaited opportunity for community pharmacists to shift from dispensing to patient care services seemed nearly at hand. many hoped that the universal PharmD, evidence supporting the positive impact of pharmacist services, and the Part D MTM benefit would create new opportunities for pharmacists to transition from a product focus to a patient focus. However, the vast majority of community pharmacists still found themselves dispensing by the end of the 2000s. Substantial progress had been made since the 1980s, but the pace of change was slower than what pharmacy’s leaders had hoped for.

## 5. 2010–Present: Post-Pharmaceutical Care Era

### 5.1. Education

By 2010, the all-PharmD requirement had been fully implemented for a decade. Instead of changes to degrees, the largest changes in pharmacist education occurred after graduation. Pursuit of residencies has increased steadily in the post-pharmaceutical care era, with the number of students pursuing residencies over this period more than doubling between 2009 and 2018 [42,43] and percent of students pursuing residencies increasing from 21.8% to 29.0% [42,43]. Residencies have become a prerequisite for entry-level clinical pharmacy jobs in inpatient settings, but community pharmacy residencies have not become the de facto standard for entry into community pharmacy practice. The number of sites remains small, and few graduating students pursue them. This may change in the future, but residencies for community pharmacists remain limited primarily to those pharmacists who intend to provide an exceptional amount of non-dispensing services [27].

### 5.2. Practice

Immunizations and patient care services have both increased in the 2010s. Community pharmacists’ immunization-related scope of practice has continued to increase and pharmacies have become an accepted place to receive an immunization. The majority of patients report feeling comfortable receiving vaccines in pharmacy settings [44], and more than 22% of all people who got vaccinated for influenza during the 2014–15 flu having received their immunization from a pharmacy or store [45]. Evidence supports the role of the pharmacist in positively impacting vaccination rates for a variety of immunizations [46], and immunizations will remain a feature of community pharmacy practice for the foreseeable future.

Other non-dispensing services have increased throughout the second decade of the 2000s, but the rate of increase has been slow. More than 20 years after the publication of his landmark paper on the opportunities and responsibilities of pharmaceutical care, Dr. Hepler referred to the idea of pharmacy as a clinical profession as a “dream deferred” when giving the 2010 Whitney Lecture [47]. This frames well the state of pharmaceutical care services in the 2010s—not dead, but not as viable as pharmacy leaders in the 1980s and 1990s had hoped. Results of the most recent pharmacy workforce survey find that pharmacists in community settings spend about an hour of each day providing patient care services not associated with dispensing [48]. This is more than past workforce surveys, but more than two-thirds of community pharmacists’ time remains spent dispensing [48], and dispensing remains the role patients perceive as most valuable [44].

Accordingly, the promise of MTM in Part D had lost some of its shine by 2010. Low beneficiary uptake and lackluster offerings resulted in the federal government efforts to strengthen requirements for offering MTM [49]. Additionally, the federal government began to include the comprehensive medication review (CMR) completion rate as a quality measure for the Medicare Stars Rating program [50]. Higher quality scores within this program result in greater marketing and revenue opportunities for Part D plan sponsors, and since the CMR completion rate measure was first introduced, completion rates have increased from 15.4% to 38.0% and 30.9% to 71.0% for standalone prescription drug plans and Medicare Advantage prescription drug plans respectively [51]. OutcomesMTM^®^, a major facilitator of pharmacist-provided MTM services, processed 2.4 million MTM claims in 2016 alone [52]. This was an increase over prior years [53], but was still less than a claim a week for the 50,000 pharmacists actively participating in the OutcomesMTM network.

One potential opportunity to expand services is through provider status in Medicare Part B Legislation that would expand billing opportunities for pharmacists in Medicare Part B was introduced during the 114th and 115th Congress but failed to pass [54]. This would create a sustainable source of revenue for non-dispensing services, but the passage of federal legislation is uncertain. More movement towards provider status has happened at the state level [55], but these efforts oftentimes expand privileges without expanding opportunities to bill insurers.

The brightest light for the post-pharmaceutical care area are the opportunities beginning to emerge for community pharmacies as partners with payers and care givers to improve medication-related quality measures and decrease total cost of care. These engagements have taken two general forms. The first is pharmacists being held to account on quality measures like medication adherence, and receiving upside and downside payment adjustment based on performance. Nearly all pharmacies in the US have the ability to support performance measurement for intermediate outcomes like medication adherence [44], and nearly 60% of 2019 Medicare beneficiaries are in a plan that has a performance-based pharmacy payment program related to quality measures [56]. The second type of engagement is contracting between community pharmacies and payers or other provider groups to share risk and provide enhanced services aimed deliberately at impacting cost and quality. These types of engagements are relatively new, but are being supported by groups such as CPESN USA [57] which brings together community pharmacies willing to provide enhanced services into clinically integrated networks able to manage populations of patients across groups of pharmacies. This approach is a step far beyond one-off pharmacy collaborations, and could finally establish a working business model for the professional service-oriented pharmacy.

## 6. Conclusions

Throughout the modern era, pharmacy has pursued higher professional standing. In the 1920s, this meant eschewing the soda fountain and front-end sales to focus on compounding and dispensing. By the 1950s, professional standing had begun to be defined more by patient care services than by simple dispensing. However, dispensing has remained stubbornly prominent in community pharmacy practice, and the opportunities to provide patient care services have not been as plentiful as hoped. Over the same period, there has been an interplay between education and practice, with education driving higher professional standing through the eventual adoption of the universal PharmD standard, and entrepreneurial pharmacists developing new, innovative practices focused on providing greater patient care services. Looking towards the future, it is hard to imagine a community pharmacist in the mid-21st century doing nothing but dispensing, but it is equally hard to imagine that dispensing would not be a part of how the average community pharmacist spends his or her day. Immunizations will almost certainly remain a common feature of pharmacy practice, as will some degree of patient care services not associated with dispensing. Evidence linking the provision of these services to reductions in healthcare spending and improvements in healthcare quality is growing. If community pharmacists can demonstrate that their services have a meaningful impact on healthcare value, it is likely their non-dispensing roles will continue to increase. Absent this evidence, one wonders how the community pharmacist in 2050 will spend their day. Nevertheless, developments in the last 100 years have created new opportunities for community pharmacists to provide patient care services not associated with dispensing, and as society evolves community pharmacy practice will continue to evolve alongside it.
